# Assessment of human health hazard associated with heavy metal accumulation in popular freshwater, coastal and marine fishes from south-west region, Bangladesh

**DOI:** 10.1016/j.heliyon.2023.e20514

**Published:** 2023-09-29

**Authors:** Anusree Biswas, Kaniz Fatema Kanon, Md. Anisur Rahman, Mohammad Shafiqul Alam, Sudipta Ghosh, Md. Almamun Farid

**Affiliations:** a Department of Fisheries and Marine Bioscience, Jashore University of Science and Technology, Jashore, 7408, Bangladesh; b Department of Genetics and Fish Breeding, Bangabandhu Sheikh Mujibur Rahman Agricultural University, Gazipur, 1706, Bangladesh

**Keywords:** Freshwater fish, Health hazard, Heavy metal, Marine fish and Multivariate approach

## Abstract

An analysis was conducted on both freshwater, coastal and marine fish species to evaluate the concentrations of heavy metals, with the aim of assessing their levels and examining the potential health risks for humans linked to the consumption of contaminated fish. This study estimate concentrations of Cr, Fe, Cu, As, Cd and Pb in 60 individuals belonging to 20 species (10 species for freshwater and another 10 for coastal and marine fishes) by using Atomic Absorption Spectrophotometer. Metal concentrations of Cr, Fe, and Pb in freshwater fishes and Cr, Fe, As, and Pb in marine fishes were exceeded the maximum allowable concentration (MAC). The Estimated Daily Intake (EDI), Average Pollution Load Index (APLI), Target Hazard Quotient (THQ), Hazard Index (HI) and Target Cancer Risk (TCR) of heavy metals were determined for the assessment of human health risk. Ranking order of the values of EDI for freshwater fishes, coastal and marine fishes were Cd > Fe > Pb > Cr > Cu > As and Cd > Fe > Pb > Cr > As > Cu. Highest APLI value of 8.14 (*Puntius ticto*) that is seriously polluted and 3.003 observed in *Otolichthoides pama* in freshwater and marine fishes, respectively. THQ_Pb_ and THQ_As_ for all the fish species were exceed the safe limit (THQ>1) suggesting potential health risk to consumers. The hazard index for both the fish samples were exceeded the USEPA (United States Environmental Protection Agency) permitted risk level (HI > 1). The target carcinogenic risk value for Cr and As were crossed the USEPA standard limit (TCR> 1E-04), which denotes that continuous consumption of studied fishes may cause health risk to the consumers. On the other hand, sensitivity analysis of freshwater, coastal and marine fishes indicates that all the metal concentrations were responsible factor for health risk.

## 1 Introduction

A metal which have an atomic number of >20 and density exceeds 5 g cm^−3^ is considered as heavy metal [1]. Throughout the earth's crust two types of heavy metals are found as essential Zn, Fe, Ca, Na, K etc. and Pb, Cd, Ni, Cr, As etc. are named as toxic heavy metal when these exceeds permissible limit. Heavy metals are irrefrangible and have noxious effect on living organisms as well as aquatic environment [2]. Heavy metals can enter rivers from various sources, including industrial discharges, agricultural runoff, and urban runoff. Industries, such as mining, metallurgy, and manufacturing, often release heavy metals as byproducts of their processes. Once released into the water, these heavy metals can be taken up by aquatic organisms, such as algae, plants, and small animals [3]. This process is known as bioaccumulation, where concentrations of the metals increase in the tissues of organisms over time. Heavy metals, as mentioned earlier, are a group of metallic elements that can be highly toxic to living organisms, including humans. Even at low concentrations, heavy metals like lead, mercury, cadmium, and arsenic can pose serious health risks. These metals are persistent in the environment and can accumulate in the soil and groundwater over time [4]. In the ecosystem, gradually the concentration of toxic metals are reaching unprecedented levels. Heavy metal cannot be destroyed because they are non-biodegradable, have tendency to bio-accumulated [5]. Bangladesh is a highly populated country and one of the most fish consuming nation in the globe, that's why capture fish are not sufficient to meet the increasing demand [6]. As a consequences, aqua farming practice is increasing day by day. In recent year, the aquaculture production is 26.38 lakh MT [7]. The use of farmed made feed is increasing dramatically to sustain the aquaculture production. In order to provide the balanced nutrition of farmed fish, different feed factories are using toxic elements in artificial feed. Due to their position at the apex of the aquatic food chain, fish have a tendency to accumulate significant quantities of metals from the surrounding water [[8], [9]]. Heavy metals are the naturally occurring elements in aquatic environment and their concentrations may commenced from natural and anthropogenic sources such as urbanization, industrialization, massive land use changes and terrestrial runoff [10]. In Chattagram coastal area of Bangladesh, the main sources of metal contamination is ship breaking activities [11]. Again, various disposable materials are being released and spilled from scrapped ships. People living in and around coastal areas, marine fishes are important sources of protein and they form a crucial part of the diet of human being. Marine fishes constitute of lower level of cholesterol. Calories and saturated fats and have digestible protein, minerals (Ca, I, K, Zn and Fe), vitamins as well as polyunsaturated fatty acids etc [12]. Comprehensively, fishes are excellent bio-marker for metal contamination because they amalgamate heavy metals in different parts of the body and occupy different trophic levels [13]. Consumption of freshwater, coastal and marine water fishes for lengthened time consequences in accumulation of heavy metal in human being and create some chronic disease [[14], [15]]. Prolonged ingestion of trace metals present in fish has been associated with a range of adverse health outcomes in humans, including compromised reproductive and hematological function, increased risk of cardiovascular ailments, neurological disorders like Parkinson's and Alzheimer's diseases [[16], [17], [18]]. The harmful impact of consuming cadmium (Cd) and lead (Pb) encompassing a wide range of health consequences, including impaired immune function, stunted fetal growth, psychological and social impairments, malnutrition-related disabilities, and an increased risk of upper gastrointestinal cancer [19]. Renal failure, constipation, infertility, tingling and liver damage are caused by Lead [20]. Cadmium toxicity can cause human physical disorder such as kidney dysfunction, hepatic dysfunction, hypertension and poor reproductive capacity [15]. Bronchial cancer caused by the absorption of Cr. Arsenic contamination in food causes vomiting, abdominal pain and severe diarrhea [21]. Several researchers have been conducted on heavy metal concentration of different fishes like accumulation of heavy metals in the freshwater fish *Pangasianodon hypophthalmus* in Mymensingh [22]. Exposure to potentially toxic elements through ingestion of canned non-alcoholic drinks [23]. Health risk assessment and quantitative source apportionment of dissolved metals in ponds [24]. Evaluation of metal accumulation in Terme River sediments using ecological indices and a bioindicator species [25], Bangladesh. Heavy metal concentrations in commercially valuable fishes with health hazard inference from Karnaphuli River [13]. Heavy metal contamination in some selected commercial fish feeds used in Bangladesh [26]. The distribution of arsenic and heavy metals and correlation among them in Groundwater of South Fukra, Gopalganj, Bangladesh [4]. Heavy metals in chrome-tanned shaving of the tannery industry are a potential hazard to the environment of Bangladesh [27]. Heavy metals contamination of river water and sediments in the mangrove forest ecosystems in Bangladesh [28]. But no research has been focused on human health risk and heavy metal concentration of freshwater, coastal and marine water fishes in south west region, Bangladesh. So, the present study (i) investigate the concentration of heavy metal (Cr, Fe, Cu, As, Cd and Pb) in different commercial freshwater, coastal and marine water fish species and (ii) determine the carcinogenic and non-carcinogenic public health risk through the taking of freshwater, coastal and marine water fish species.

## 2 Materials and methods

### 2.1 Sample collection and preparation

**Fig. 1:**
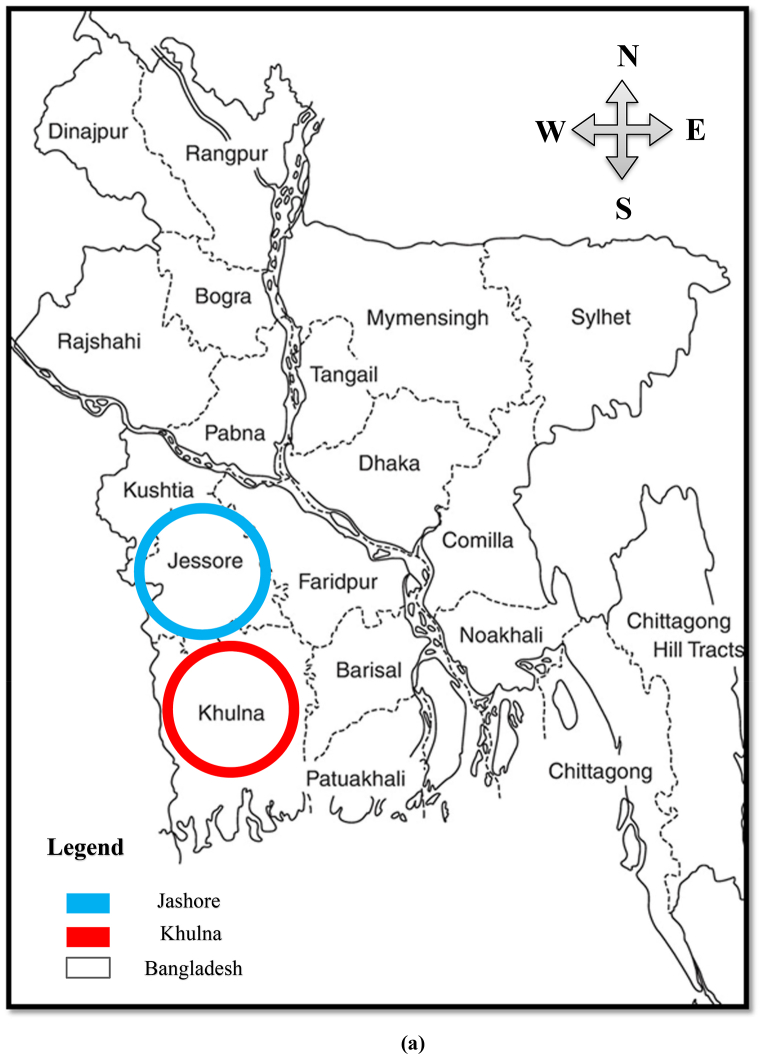

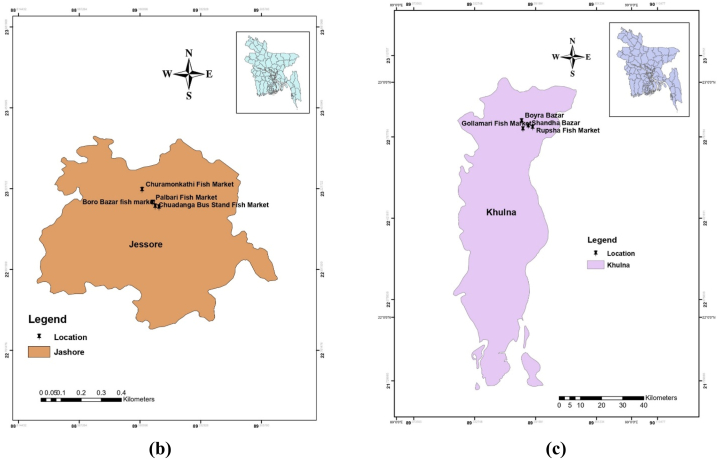
The map of study area in Bangladesh, (a) studied fish market in Jashore (b) and studied fish market in Khulna (c).

A total of 60 individuals belonging to 20 species, constituting 10 for freshwater and another 10 for coastal and marine water fish species. Fish samples were collected from 8 different fish markets in south-west region of Bangladesh (Fig. 1a), among them four markets are located in Jashore (Bora bazar, Palbari bazar, Chuadanga bus stand bazar and Churamonkathi fish market) (Fig. 1b) and other 4 in Khulna district (Rupsha whole sale fish market, Boyra bazar, Shandha bazar and Gollamari fish market) (Fig. 1c). The name of six heavy metals estimated in these samples were Cupper (Cu), Arsenic (As), Iron (Fe), Lead (Pb), Chromium (Cr), and Cadmium (Cd).

Prior to sampling, the fish samples underwent measurements of weight and body length. Subsequently, the samples were carefully washed three times using clean tap water and deionized water, followed by drying with tissue paper. To facilitate analysis, the fish samples were then divided into three cross-sectional slices, each approximately 2.5 cm thick. The first slice was positioned just behind the pectoral fins, the second slice was taken halfway between the first slice and the vent, and the third slice was obtained just behind the vent. Edible parts of all samples were extracted except inedible part (fins, scales, tail, bone, gut etc.). Then samples were dried at 80 °C in Fisheries and Marine Bioscience laboratory oven until fish attained a constant weight as well as ground the samples properly to form powder by using agnate motor.

### 2.2 Reagent preparation

To assess targeted elements from the samples, all standard solutions were prepared with analytical reagent-grade chemicals and ultra-pure water. All standard solutions were supplied by Merck Germany with 99.9% purity level. 2% HNO_3_ solution have been used to rinse and soak the plastic and glassware for overnight.

### 2.3 Sample digestion

In the present investigation, an acid digestion method in which, HNO_3_ and H_2_O_2_ were used [29]. In this method 1.0 g fish sample was weighed and taken into the digestion tube, then 10 ml of HNO_3_ were added in the tube. The samples were placed in a hot plate and heated to 95 ± 5 °C for 10 min and kept to cool them. Then, samples were placed in a digestion chamber and 5 ml of HNO_3_ was added as well as evaporated off at 100 °C for 2 more hours. Again, samples were cooled, and 3 ml of 30% H_2_O_2_ with 2 ml of double distilled water were added. Then the samples were kept in digestion block for 2 more hours at 100 °C. The digestion is complete when the final solution becomes clear. Finally, all the digests were filtered with Whitman filter paper (Size-40) and stored into 50 ml plastic container [30].

### 2.4 Analytical methods and quality control

The atomic absorption spectrophotometer (AAS) with a digital readout system (Model AA-6800, Shimadzu Corporation, Japan) was utilized to analyze the concentration of Cr, Fe, Cu, As, Cd and Pb in the digested solutions. For determining As concentrations, hydride vapor generators (HVG) were employed in conjunction with the flame AAS (FAAS) systems. The calibration standards for the instrument were prepared by diluting supplied standards (1000 mg/l) from Wako Pure Chemical Industry Ltd., Japan. To assess the method's accuracy, blanks and a certified reference material MA-A-2 [fish-flesh standard from International Atomic Energy Agency (IAEA), Vienna] were analyzed using the same procedure employed for fish samples. The mean recoveries of the analyzed metals ranged from 95% to 104%, demonstrating a favorable agreement between the certified and measured values.

### 2.5 Statistical analysis

For the analysis of data in the current study, the SPSS V.16.0 software (SPSS, USA) was utilized. SPSS was employed to calculate the standard deviation and average values of the studied samples. To facilitate a clearer understanding, tables and graphical presentations were created using MS Excel (Version 2013).

### 2.6 Health risk assessment

#### 2.6.1 Estimated daily intake (EDI) of metals

In order to assess the daily average intake of heavy metals into the body of a consumer, taking into account their specific body weight, the Estimated Daily Intake (EDI) was measured. The EDI for the investigated metals in freshwater, coastal and marine fish species was determined by considering the metal concentration in these fish, along with the average daily consumption of an adult and their average body weight.

The EDI was determined by using following formula [31] in Eq. (1) as below:

(1) EDI = (FIR × C) / BW

Where, FIR=Fish ingestion rate; on average an adult FIR 59.91 g/person/day; C = Heavy metal concentration in fishes (mg/kg, dry weight); and BW = average body weight (60 kg).

#### 2.6.2 Pollution load evaluation method

The APLI approach is used to assess the harmful effects of different metals on exposed [32] freshwater, coastal and marine fishes, the following formula is used in Eq. (2).

(2) $$APLI=\frac{1}{n}\;\sum_{n=1}^{n}\frac{Ci}{Si}$$

The APLI (Average Pollution Load Index) is a method used to assess the contamination of metals and metalloids in fish samples. It is calculated based on the concentrations of different metals and metalloids measured in the samples, their maximum admissible concentrations (MAC), and the number of metals and metalloids (n). Ci is the mean concentration of metal or metalloid in the fish samples and Si is the maximum admissible concentration (MAC) of metal or metalloid. Based on the calculated APLI value, the contamination level in the fish samples can be categorized into different pollution levels- APLI <0.1: Unpolluted, 0.1–0.2: Micro-pollution, 0.2–0.5: Lightly polluted, 0.5–0.7: Moderately polluted, 0.7–1.0: Heavily polluted and APLI >1.0: Seriously polluted. So, the greater the APLI value, the more polluted the fish samples.

#### 2.6.3 Target hazard quotient

The estimation of the risk level of non-carcinogenic effects resulting from heavy metal exposure is conducted using the Target Hazard Quotient (THQ). The THQ values are determined by employing the following formula in Eq. (3), as per the [29] standard assumption:

(3) $$\text{THQ}=\frac{\text{EFr}\times \text{ED}\times \text{FIR}\times C}{\text{RfD}\times \text{BW}\times \text{AT}}\times 10^{-3}$$

In this equation, THQ represents the target hazard quotient. EFr corresponds to the exposure frequency (365 days/year), ED denotes the exposure duration (70 years), FIR signifies the fish ingestion rate (average adult consumption rate of 59.91 g/person/day), C represents the concentration of heavy metals in fishes (mg/kg, dry weight), BW stands for the average body weight (60 kg), AT denotes the average exposure time for non-carcinogens (EFr × ED) (365 days/year for 70 years), and RfD represents the oral reference doses specified as 0.003, 0.0005, 1.5, 0.04, and 0.0085 mg/kg/day for As, Cd, Cr, Cu, and Pb, respectively [33].

#### 2.6.4 Hazard index

When an individual is exposed to multiple toxicants, a cumulative effect known as the Hazard Index (HI) occurs. The HI is determined through a mathematical calculation in which the THQ values of the analyzed fish samples are summed together. This calculation, which involves adding the individual THQ values, follows the methodology outlined [33]. In the present research study, the HI was estimated using the following approach in Eq. (4).

(4) HI = THQ (As) + THQ (Cd) + THQ (Cr) + THQ (Fe) + THQ (Cu) + THQ (Pb).

When the Hazard Index (HI) exceeds 1, it indicates that the non-carcinogenic risk is elevated for the exposed group of individuals [34].

#### 2.6.5 Target cancer risk (TCR)

The estimation of cancer risk involves calculating the likelihood of an individual developing cancer over their lifetime as a result of exposure to potential carcinogens [34]. The target cancer risk (TCR) is determined by multiplying the daily dose by the cancer slope (CSF), which is derived from the dose-response curve of the ingested poison. This formula follows the methodology outlined by Ref. [29] in Eq. (5).

(5) $$\text{TCR}=\frac{\text{EFrxEDxFIRXCSF}}{\text{BWXAT}}X10^{-3}$$

## 3 Results and discussion

### 3.1 Metal concentration in freshwater fish species

The research work was conducted to evaluate the metal concentration in freshwater fish muscles. The ranking order of average concentration of toxic metal in different fish species were Fe > Pb > Cr > Cu > As > Cd (Table 1). The average concentration of Cu was 1.54 mg/kg, whereas the highest value was found in *Labeo bata* (2.77 mg/kg) and lowest for *Channa punctatus* (0.75 mg/kg). Our result showed that Cu concentration in all freshwater fish samples were below the permissible limit 4.5 mg/kg [35]. The average Cu concentration of fish species was 4.71 mg/kg in western part of Bangladesh [36]. The Cu concentration was 7.089 mg/kg [37]. The present research work revealed that *Orechromis nilotichus* showed the highest mean value of As was 2.17 mg/kg (Table 1), which cross the FAO standard guideline (1.0 mg/kg) expressing that Tilapia fish species were contaminated by As. Arsenic contamination in food causes vomiting, abdominal pain and severe diarrhea [21]. The highest mean concentration of As was 2.13 mg/kg in Karnaphuli River, Bangladesh [13]. In this study the maximum and minimum mean concentration of Fe was detected (152.4 mg/kg) and (22.68 mg/kg) in *Mystus tengera* and *Hypophthalmichthys molitrix,* respectively (Table 1). Highest Fe concentration in seafood (272.7 mg/kg) from Saint Martin Island, Bangladesh [15].

**Table 1:** Heavy metal concentrations (mg/kg dw) in 10 freshwater fish species sampled from Jashore and Khulna district, Bangladesh (mean ± SD).

English name	Scientific name	Cu	As	Fe	Pb	Cr	Cd
**Sarpunti**	*Puntius sarana*	1.50 ± 0.06	0.11 ± 0.02	30.25 ± 1.95	3.89 ± 0.75	3.30 ± 0.37	0.07 ± 0.02
**Bata**	*Labeo bata*	2.77 ± 0.38	0.23 ± 0.02	46.20 ± 1.65	2.99 ± 0.05	3.92 ± 0.28	0.09 ± 0.009
**Tangra**	*Mystus tengera*	2.60 ± 0.18	0.13 ± 0.01	152.4 ± 1.48	9.10 ± 0.18	10.62 ± 1.003	0.16 ± 0.01
**Silver carp**	*Hypophthalmichthys molitrix*	0.97 ± 0.13	1.13 ± 0.04	22.68 ± 1.40	8.74 ± 0.23	3.73 ± 0.21	0.10 ± 0.006
**Punti**	*Puntius ticto*	2.73 ± 0.30	0.72 ± 0.10	75.20 ± 2.40	24.41 ± 0.14	14.33 ± 0.98	0.32 ± 0.02
**Pangus**	*Pangasius pangasius*	0.90 ± 0.05	0.09 ± 0.01	26.82 ± 0.69	7.37 ± 0.19	1.15 ± 0.15	0.05 ± 0.008
**Tilapia**	*Orechromis nilotichus*	1.37 ± 0.04	2.17 ± 0.10	36.64 ± 1.73	7.33 ± 0.14	2.33 ± 0.37	0.04 ± 0.007
**Pabda**	*Ompok bimaculatus*	0.78 ± 0.05	0.13 ± 0.01	26.74 ± 0.83	8.25 ± 0.18	1.39 ± 0.19	0.02 ± 0.005
**Bele**	*Glossogobius giuris*	0.99 ± 0.06	0.42 ± 0.06	32.59 ± 0.98	17.15 ± 0.36	4.87 ± 0.16	0.09 ± 0.02
**Taki**	*Channa punctatus*	0.75 ± 0.06	0.23 ± 0.01	59.63 ± 3.34	7.13 ± 0.15	2.38 ± 0.16	0.021 ± 0.006
**Maximum allowable Conc. (MAC) (FAO, WHO, 2002) 4.5 1.0 0.5 1.0 0.1**							

Note: Maximum Allowable Concentration (MAC), Food and Agricultural Organization (FAO), and World Health Organization (WHO).

Among the studied fish species the highest average concentration of Pb (24.41 mg/kg) in *Punctius ticto,* whereas, *Labeo bata* showed the lowest average concentration (2.99 mg/kg). The average value of Lead for all studied freshwater fish species was 9.64 mg/kg (Table 1), higher than the FAO standard guideline (0.5 mg/kg). The highest mean concentration of Pb was 0.240 mg/kg in *Siniperca chuatsi* at Dongting Lake, China [38]. In our study, Cr had the highest mean concentration in *Punctius ticto* with 14.33 mg/kg and lowest for *Pangasius pangasius* was 1.15 mg/kg. In addition, the average Cr concentration of all freshwater fishes was 4.80 mg/kg which exceeded the FAO limit (1.0 mg/kg). The average concentration 2.23 mg/kg Cr in the fish species of Dhaleshwari River, Bangladesh [39]. In this research work showed that the average Cd concentration was 0.09 mg/kg. The concentration of Cd in all sample were within the limit of FAO guideline (0.1 mg/kg) except *Punctius ticto* (0.32 mg/kg).

### 3.2 Metal concentration in coastal and marine fish species

The concentrations of heavy metal in 10 coastal and marine fish species are given in Table 2. The ranking order of average concentration of toxic metal in different coastal and marine fish species were Fe > Pb > Cr > As > Cu > Cd (Table 2) and the average values were as follows Fe (34.12 mg/kg), Pb (9.09 mg/kg), Cr (2.89 mg/kg), As (2.64 mg/kg), Cu (1.43 mg/kg) and Cd (0.09 mg/kg), respectively. The highest value of Cu concentration in *Hilsa ilisha* fish species was 5.70 mg/kg, which exceeds the international limit of 4.5 mg/kg [35]. Mean value of Cu concentrations (1.43 mg/kg) for all coastal and marine fishes. The average Cu concentration of marine fish species was 3.89 mg/kg in China that was observed [37]. The average Cu concentrations ranged from 0.364 to 0.403 mg/kg in *Oncorhynchus mykiss* [40]. In present research work, the average value of As was 2.64 mg/kg which cross the FAO standard guideline (1.0 mg/kg). Iron is an essential metal for fish metabolism [14]. In the studied marine fish sample, the mean concentration (34.12 mg/kg) of Iron, in which, *Hilsa ilisha* showed the maximum mean value 74.78 mg/kg. The maximum mean concentration (16.50 mg/kg) of Fe in *Sparus aurata* [41]. European anchovy (21.36 mg/kg) had the highest mean concentration [42]. The highest (9.09 mg/kg) and lowest (6.14 mg/kg) mean concentrations of Pb were in *Otolithoides pama and Mugil cephalus,* respectively. The average concentrations (7.28 mg/kg) of Pb for all the studied fishes were exceed the FAO recommended safe value (0.5 mg/kg). In *Bagarius bagarius,* the highest mean concentration of Pb was 1.87 mg/kg documented at Dhaleshwari River, Bangladesh [39]. The highest concentration (6.68 mg/kg) of Cr was detected in *Escualosa thoracata* fish and the lowest in *Nemipterus virgatus* (0.98 mg/kg). The standard value of Cr 1.0 mg/kg was given by FAO, which crossed the mean concentration (2.89 mg/kg) of Cr in present study. Concentration of Cr was 0.623 mg/kg in *Labeo rohita* [5]. Cadmium compounds are carcinogenic to human health, it is an extremely toxic pollutants. Long term exposure can cause kidney, liver and respiratory organ damage. Highest average Cd concentration (0.24 mg/kg) was found in *Hilsa ilisha* fish. Cadmium Concentration follows the order as *Hilsa ilisha* > *Escualosa thoracata* > *Liza parsia* > *Thunnus thynnus* > *Nemipterus virgatus* > *Polynemus paradiseus* > *Pampus chinensis* > *Mugil cephalus*. The average value (0.09 mg/kg) of Cr exceeds the permissible limit (0.05 mg/kg). The Cadmium concentration was 0.88 μg/g of fishes in Nepal [43].

**Table 2:** Heavy metal concentrations (mg/kg dw) in 10 coastal and marine fish species sampled from Jashore and Khulna district, Bangladesh (mean ± SD).

English name	Scientific name	Cu	As	Fe	Pb	Cr	Cd
**Parse**	*Liza parsia*	1.67 ± 0.04	4.24 ± 0.03	28.60 ± 1.12	6.62 ± 0.08	3.83 ± 0.12	0.12 ± 0.004
**Poa**	*Otolithoides pama*	1.16 ± 0.07	2.27 ± 0.06	32.99 ± 1.09	9.09 ± 0.01	5.19 ± 0.33	0.12 ± 0.007
**Marine mola**	*Escualosa thoracata*	1.17 ± 0.04	2.10 ± 0.05	35.21 ± 2.27	7.76 ± 0.01	6.68 ± 0.36	0.15 ± 0.007
**Colombo Ilish**	*Hilsa ilisha*	5.70 ± 0.25	4.05 ± 0.03	74.78 ± 2.62	7.76 ± 0.06	4.35 ± 0.29	0.24 ± 0.01
**Golden threadfin bream**	*Nemipterus virgatus*	0.59±±0.03	2.05 ± 0.11	19.39 ± 1.39	7.93 ± 0.15	0.98 ± 0.13	0.08 ± 0.01
**Tapshe**	*Polynemus paradiseus*	1.59 ± 0.08	3.95 ± 0.13	25.52 ± 0.58	7.57 ± 0.12	1.28 ± 0.17	0.06 ± 0.008
**Tulordandi**	*Sillaginopsis panijus*	0.59 ± 0.05	1.75 ± 0.04	19.38 ± 1.56	6.70 ± 0.15	1.28 ± 0.2	0.05 ± 0.007
**Rupchanda**	*Pampus chinensis*	0.46 ± 0.04	1.61 ± 0.13	24.37 ± 1.20	6.67 ± 0.23	2.13 ± 0.33	0.05 ± 0.01
**Mullet**	*Mugil cephalus*	0.98 ± 0.06	1.99 ± 0.04	20.94 ± 1.76	6.14 ± 0.19	1.36 ± 0.19	0.02 ± 0.005
**Tuna**	*Thunnus thynnus*	0.39 ± 0.05	2.39 ± 0.08	59.97 ± 2.39	6.56 ± 0.11	1.79 ± 0.10	0.09 ± 0.008
**Maximum allowable Conc. (MAC) (FAO, WHO, 2002)**		4.5	1.0		0.5	1.0	0.1

Note: Maximum Allowable Concentration (MAC), Food and Agricultural Organization (FAO), and World Health Organization (WHO).

### 3.3 Evaluation of human health risk

#### 3.3.1 Estimation of daily intake of heavy metals from fishes

The utilization of a dietary exposure method serves as a prevalent and efficient approach to estimate the dietary patterns of the population and provide crucial insights into the exposure levels [39]. This study evaluates the dietary exposure of heavy metals through the ingestion of freshwater, coastal and marine fishes in adult people. In the present study, The EDI value for the mean concentration of every metal in fish species was calculated through the consumption of 59.91 g fish per day by an adult people which having bodyweight 60 kg [36]. The EDI values of the studied metals from the ingestion of freshwater fish are organized in Table 3. Ranking order of the values of EDI from each metal in freshwater fishes is as follows: Cd > Fe > Pb > Cr > Cu > As. Table 4, represents the estimated daily intake (EDI) of metals and metalloids average concentrations in coastal and marine fish samples. The values of EDI of individual metal from coastal and marine fishes followed the descending order of Cd > Fe > Pb > Cr > As > Cu. Obtained EDI value of coastal and marine fishes are less than the EDI value of freshwater fishes. In this study, mean EDI value for Cadmium is higher for both freshwater, coastal and marine fishes. Whereas, EDI value for Arsenic is lower for freshwater fishes and Cupper is lower for coastal and marine fishes.

**Table 3:** Estimated daily intake (EDI) (mg/kg Bw/day) of metals due to consumption of freshwater fishes.

Scientific name	Cu	As	Fe	Pb	Cr	Cd
*Puntius sarana*	1.50 × 10^−03^	1.18 × 10^−04^	3.02 × 10^−02^	3.89 × 10^−03^	3.30 × 10^−03^	2.55 × 10^−01^
*Labeo bata*	1.50 × 10^−03^	2.32 × 10^−04^	4.61 × 10^−02^	2.99 × 10^−03^	3.92 × 10^−03^	3.59 × 10^−01^
*Mystus tengera*	2.60 × 10^−03^	1.37 × 10^−04^	1.52 × 10^−01^	9.09 × 10^−03^	1.06 × 10^−02^	5.79 × 10^−01^
*Hypophthalmichthys molitrix*	9.70 × 10^−04^	1.13 × 10^−03^	2.26 × 10^−02^	8.73 × 10^−03^	3.73 × 10^−03^	3.76 × 10^−01^
*Puntius ticto*	2.74 × 10^−03^	7.25 × 10^−04^	7.51 × 10^−02^	2.44 × 10^−02^	1.43 × 10^−02^	1.15 × 10^+00^
*Pangasius pangasius*	9.07 × 10^−04^	9.94 × 10^−05^	2.68 × 10^−02^	7.36 × 10^−03^	1.15 × 10^−03^	1.93 × 10^−01^
*Orechromis nilotichus*	1.37 × 10^−03^	2.17 × 10^−03^	3.66 × 10^−02^	7.32 × 10^−03^	2.33 × 10^−03^	1.65 × 10^−01^
*Ompok bimaculatus*	7.84 × 10^−04^	1.40 × 10^−04^	2.67 × 10^−02^	8.24 × 10^−03^	1.39 × 10^−03^	7.89 × 10^−01^
*Glossogobius giuris*	9.92 × 10^−04^	4.24 × 10^−04^	3.25 × 10^−02^	1.71 × 10^−02^	4.86 × 10^−03^	3.44 × 10^−01^
**Average EDI**	1.41 × 10^−03^	5.42 × 10^−04^	5.08 × 10^−02^	9.63 × 10^−03^	4.80 × 10^−03^	3.58 × 10^−01^

**Table 4:** Estimated daily intake (EDI) (mg/kg Bw/day) of metals due to consumption of coastal and marine fishes.

Scientific name	Cu	As	Fe	Pb	Cr	Cd
*Liza parsia*	1.67 × 10^−03^	4.24 × 10^−03^	2.86 × 10^−02^	6.61 × 10^−03^	3.83 × 10^−03^	4.35 × 10^−01^
*Otolithoides pama*	1.17 × 10^−03^	2.27 × 10^−03^	3.29 × 10^−02^	9.00 × 10^−03^	5.18 × 10^−03^	4.15 × 10^−01^
*Escualosa thoracata*	1.17 × 10^−03^	2.10 × 10^−03^	3.52 × 10^−02^	7.75 × 10^−03^	6.67 × 10^−03^	5.51 × 10^−01^
*Hilsa ilisha*	5.70 × 10^−03^	4.05 × 10^−03^	7.47 × 10^−02^	7.75 × 10^−03^	4.35 × 10^−03^	8.59 × 10^−01^
*Nemipterus virgatus*	5.94 × 10^−04^	2.05 × 10^−03^	1.94 × 10^−02^	7.93 × 10^−03^	9.79 × 10^−04^	3.10 × 10^−01^
*Polynemus paradiseus*	1.59 × 10^−03^	3.95 × 10^−03^	2.55 × 10^−02^	7.56 × 10^−03^	1.27 × 10^−03^	2.23 × 10^−01^
*Sillaginopsis panijus*	5.94 × 10^−04^	1.76 × 10^−03^	1.94 × 10^−02^	6.70 × 10^−03^	1.29 × 10^−03^	1.94 × 10^−01^
*Pampus chinensis*	4.61 × 10^−04^	1.62 × 10^−03^	2.43 × 10^−02^	6.67 × 10^−03^	2.12 × 10^−03^	1.87 × 10^−01^
*Mugil cephalus*	9.87 × 10^−04^	1.99 × 10^−03^	2.09 × 10^−02^	6.14 × 10^−03^	1.36 × 10^−03^	8.62 × 10^−02^
*Thunnus thynnus*	3.97 × 10^−04^	2.39 × 10^−03^	5.99 × 10^−02^	6.56 × 10^−03^	1.79 × 10^−03^	3.30 × 10^−01^
**Average EDI**	1.43 × 10^−03^	2.64 × 10^−03^	3.41 × 10^−02^	7.27 × 10^−03^	2.88 × 10^−03^	3.59 × 10^−01^

### 3.4 Pollution evaluation indices

**Fig. 2:**
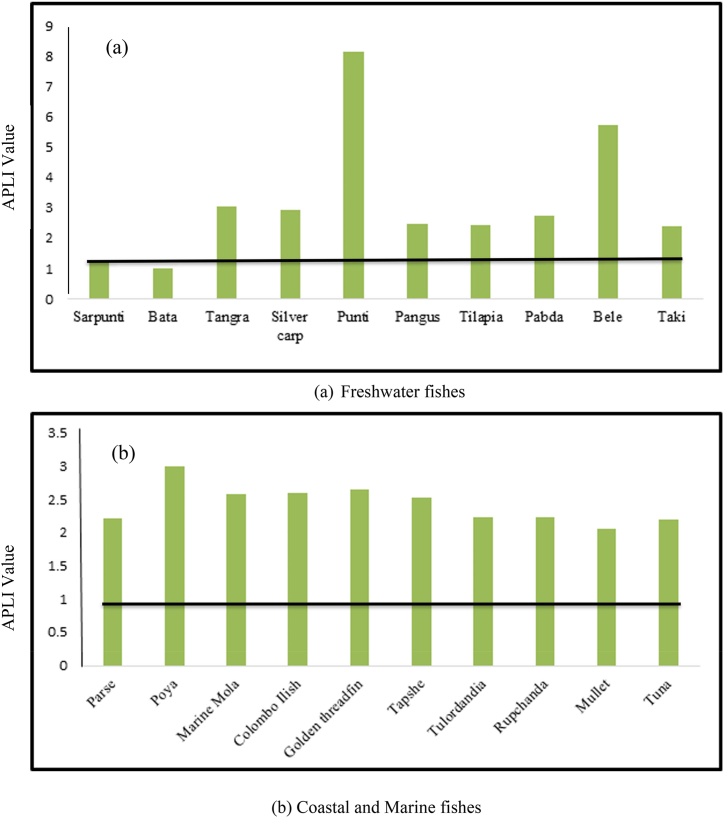
Average pollution load indices (APLI) value in freshwater (a), coastal and marine (b) fish species in the study area.

In present research work, Average pollution load indices (APLI) is use to determine the contamination level in freshwater, coastal and marine fish samples. Estimated APLI values for both freshwater, coastal and marine fishes are ranges from 0.998 to 8.14 and 2.04 to 3.003, respectively (Fig. 2 a,b). Highest APLI value of 3.003 and 8.14 observed in *Otolithoides pama* (marine fish) (Fig. 2,b) and *Punctius ticto* (freshwater fish) (Fig. 2,a). The highest APLI value found in *Channa punctata* (2.78) and *Oreochromis mossambicus* (2.31) [36]. The APLI value (0.998) of *Labeo bata* species indicates that it is unpolluted from the studied samples. Where, as the APLI value (8.14) of *Punctius ticto* showed that it is seriously polluted in this study.

### 3.5 Non-carcinogenic health risk estimation of freshwater fishes

**Fig. 3:**
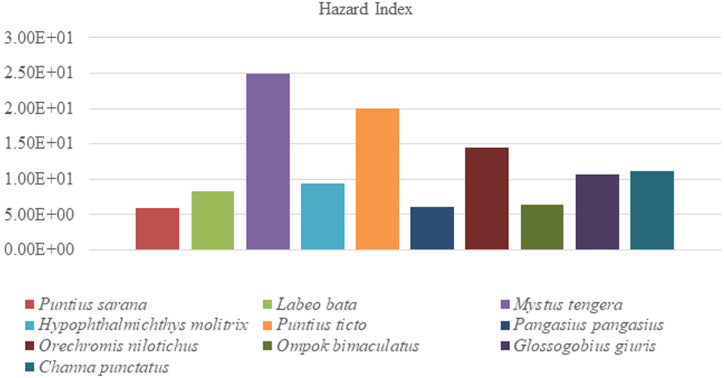
Hazard Index (HI) for six heavy metals in studied freshwater fish species.

In our present findings, the ascending ranking of THQ for all the heavy metals were as follows- Fe < Pb < As < Cd < Cr < Cu (Table 5) in freshwater fishes. The results showed that THQ_Fe_, THQ_Pb_, THQ_As_ and THQ_Cd_ for all the fish species were exceeded the safe limit (THQ>1) but, THQ_Cr,_ THQ_Cu_ for all the fish species were still remain in recommended level (THQ<1). Too much consumption of Lead, Arsenic and Cadmium contaminated fish were deleterious to the consumers. The combined effect of all heavy metals for freshwater fishes was also determined during study period. The present output suggested that the additive effect of HI showed descending manner of *Mystus tengra* > *Puntius ticto* > *Oreochromis niloticus* > *Channa punctatus* > *Glossogobius giuris* > *Hypophthalmichthys molitrix* > *Labeo bata* > *Ompok bimaculatus* > *Pangasius pangasius* > *Puntius sarana.* Hazard Index was higher for *Mystus tengra* (2.49E+01) and lower for *Puntius sarana* (5.86E+00) (Fig. 3). The combined impact of all the heavy metal levels was higher than the accepted limit (HI > 1), which denotes that consumption of those contaminated fishes were hazardous to human health.

**Table 5:** Target hazard quotient (THQ) of metals due to consumption of freshwater fishes.

English name	Scientific name	THQ-Cu	THQ-As	THQ-Fe	THQ-Pb	THQ-Cr	THQ-Cd	HI(THQ)
Sarpunti	*Puntius sarana*	3.76 × 10^−02^	3.94 × 10^−01^	4.32 × 10^+00^	9.72 × 10^−01^	2.20 × 10^−03^	1.41 × 10^−01^	5.86 × 10^+00^
Bata	*Labeo bata*	3.76 × 10^−02^	7.74 × 10^−01^	6.59 × 10^+00^	7.48 × 10^−01^	2.61 × 10^−03^	2.00 × 10^−01^	8.35 × 10^+00^
Tangra	*Mystus tengera*	6.50 × 10^−02^	4.58 × 10^−01^	2.17 × 10^+01^	2.27 × 10^+00^	7.07 × 10^−03^	3.22 × 10^−01^	2.49 × 10^+01^
Silver carp	*Hypophthalmichthys molitrix*	2.43 × 10^−02^	3.76 × 10^+00^	3.24 × 10^+00^	2.18 × 10^+00^	2.49 × 10^−03^	2.09 × 10^−01^	9.42 × 10^+00^
Punti	*Punctius ticto*	6.84 × 10^−02^	2.42 × 10^+00^	1.07 × 10^+01^	6.09 × 10^+00^	9.54 × 10^−03^	6.40 × 10^−01^	2.00 × 10^+01^
Pangus	*Pangasius pangasius*	2.27 × 10^−02^	3.31 × 10^−01^	3.83 × 10^+00^	1.84 × 10^+00^	7.68 × 10^−04^	1.07 × 10^−01^	6.13 × 10^+00^
Tilapia	*Orechromis nilotichus*	3.43 × 10^−02^	7.25 × 10^+00^	5.23 × 10^+00^	1.83 × 10^+00^	1.56 × 10^−03^	9.17 × 10^−02^	1.44 × 10^+01^
Pabda	*Ompok bimaculatus*	1.96 × 10^−02^	4.65 × 10^−01^	3.81 × 10^+00^	2.06 × 10^+00^	9.28 × 10^−04^	4.38 × 10^−02^	6.40 × 10^+00^
Bele	*Glossogobius giuris*	2.48 × 10^−02^	1.41 × 10^+00^	4.65 × 10^+00^	4.28 × 10^+00^	3.24 × 10^−03^	1.91 × 10^−01^	1.06 × 10^+01^
Taki	*Channa punctatus*	1.89 × 10^−02^	7.98 × 10^−01^	8.51 × 10^+00^	1.78 × 10^+00^	1.58 × 10^−03^	4.39 × 10^−02^	1.11 × 10^+01^
Total		3.53 × 10^−01^	1.81 × 10^+01^	7.26 × 10^+01^	2.41 × 10^+01^	3.20 × 10^−02^	1.99 × 10^+00^	1.17 × 10^+02^

### 3.6 Non-carcinogenic health risk estimation of coastal and marine fishes

**Fig. 4:**
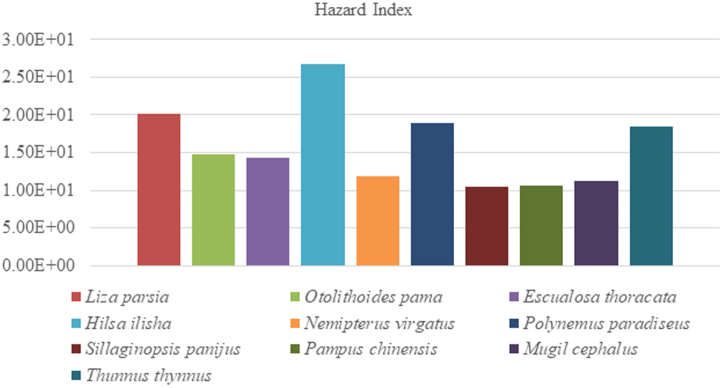
Hazard Index (HI) for six heavy metals in studied coastal and marine fish species.

Determined THQ for each heavy metal from the consumption of coastal and marine fishes are showed in Table 6. In the present study, the value of THQ's of each heavy metal followed the descending order of As > Fe > Pb > Cd > Cu > Cr. The results showed that the THQ values of Cu, Cr were below 1 and As, Fe, Pb, Cd were above 1. The THQ values of Cu, Cr were not exceeded the recommended level (THQ<1), that indicates no potential health risk for human. Target Hazard Quotient (THQ) > 1 is an indication of probable health hazard for human being. The THQ values for As, Fe, Pb and Cd showed an adverse health effect to consumers. Therefore, the additive effect of six heavy metal on human body was evaluated by calculating the value of HI. The descending ranking of HI for coastal and marine fish species were *Hilsa ilisha* > *Liza parsia > Polynemus paradiseus > Thunnus thynnus > Otolithoides pama > Escualosa thoracata > Nemipterus virgatus > Mugil cephalus > Pampus chinensis > Sillaginopsis panijus.* In Table 6, our research findings recommended that the combined effect of all heavy metals was still higher than the safe limit (HI > 1). The HI index was highest for *Hilsa ilisha* (2.67E+01) and lowest for *Sillaginopsis panijus* (1.04E-01) (Fig. 4). The HI index was highest for *L. mystaceus* (7.06E+01) in Tigris River [40]. In Bosnia and Harzegovina, the Hazard Index in Tuna fish (0.92) and Mackerel (0.84) [44]. Daily intake of this species are harmful to consumers.

**Table 6:** Target hazard quotient (THQ) of metals due to consumption of coastal and marine fishes.

Scientific name	THQ-Cu	THQ-As	THQ-Fe	THQ-Pb	THQ-Cr	THQ-Cd	HI (THQ)
*Liza parsia*	4.18 × 10^−02^	1.41 × 10^+01^	4.08 × 10^+00^	1.65 × 10^+00^	2.55 × 10^−03^	2.42 × 10^−01^	2.02 × 10^+01^
*Otolithoides pama*	2.92 × 10^−02^	7.57 × 10^+00^	4.71 × 10^+00^	2.25 × 10^+00^	3.46 × 10^−03^	2.30 × 10^−01^	1.48 × 10^+01^
*Escualosa thoracata*	2.93 × 10^−02^	7.00 × 10^+00^	5.02 × 10^+00^	1.94 × 10^+00^	4.45 × 10^−03^	3.06 × 10^−01^	1.43 × 10^+01^
*Hilsa ilisha*	1.42 × 10^−01^	1.35 × 10^+01^	1.07 × 10^+01^	1.94 × 10^+00^	2.90 × 10^−03^	4.77 × 10^−01^	2.67 × 10^+01^
*Nemipterus virgatus*	1.49 × 10^−02^	6.85 × 10^+00^	2.77 × 10^+00^	1.98 × 10^+00^	6.53 × 10^−04^	1.72 × 10^−01^	1.18 × 10^+01^
*Polynemus paradiseus*	3.98 × 10^−02^	1.32 × 10^+01^	3.64 × 10^+00^	1.89 × 10^+00^	8.50 × 10^−04^	1.24 × 10^−01^	1.89 × 10^+01^
*Sillaginopsis panijus*	1.49 × 10^−02^	5.86 × 10^+00^	2.76 × 10^+00^	1.67 × 10^+00^	8.58 × 10^−04^	1.08 × 10^−01^	1.04 × 10^+01^
*Pampus chinensis*	1.15 × 10^−02^	5.39 × 10^+00^	3.48 × 10^+00^	1.67 × 10^+00^	1.42 × 10^−03^	1.04 × 10^−01^	1.06 × 10^+01^
*Mugil cephalus*	2.47 × 10^−02^	6.65 × 10^+00^	2.99 × 10^+00^	1.53 × 10^+00^	9.04 × 10^−04^	4.79 × 10^−02^	1.12 × 10^+01^
*Thunnus thynnus*	9.93 × 10^−03^	7.98 × 10^+00^	8.56 × 10^+00^	1.64 × 10^+00^	1.20 × 10^−03^	1.84 × 10^−01^	1.84 × 10^+01^
**Total**	3.58 × 10^−01^	8.81 × 10^+01^	4.87 × 10^+01^	1.82 × 10^+01^	1.92 × 10^−02^	1.99 × 10^+00^	1.57 × 10^+02^

### 3.7 Carcinogenic risk characterization of freshwater fishes

Table 7, displays the carcinogenic risk associated with exposure to heavy metals from the investigated freshwater fish species. The present research study considered the availability of carcinogenic potency slope factors for As, Pb, Cr, and Cd. Generally, CR > 10^−4^ is considered as unacceptable, 10^−6^<CR < 10^−4^ is considered as acceptable range and CR < 10^−6^ considered as negligible [29]. TCR values for As and Cr ranged from 1.49 × 10^−04^ to 3.26 × 10^−03^ and 5.76 × 10^−04^ to 7.15 × 10^−03^ respectively, suggesting all of the freshwater fish species have As and Cr associated cancer risk. On the other hand, the TCR values for Pb and Cd varied from 2.54 × 10^−05^ to 2.07 × 10^−04^ and 8.33 × 10^−06^ to 1.22 × 10^−04^ respectively, indicating that all the fish species are safe from Pb and Cd related cancer risk. In case of total target carcinogenic risk (TTCR) for freshwater fishes are ranged from 8.08 × 10^−04^ to 8.57 × 10^−03^ which denotes that long-term consumption of these fish species have cancer risk to the consumers. The CR value for As ranged from 1.15 × 10^−05^ to 7.36 × 10^−06^ in common carp which suggested that there was low carcinogenic risk [45].

**Table 7:** Target carcinogenic risk (TCR) of metals due to consumption of freshwater fishes.

English name	Scientific name	TCR-As	TCR-Pb	TCR-Cr	TCR-Cd	TTCR
Sarpunti	*Puntius sarana*	1.77 × 10^−04^	3.31 × 10^−05^	1.65 × 10^−03^	2.69 × 10^−05^	1.89 × 10^−03^
Bata	*Labeo bata*	3.48 × 10^−04^	2.54 × 10^−05^	1.96 × 10^−03^	3.79 × 10^−05^	2.37 × 10^−03^
Tangra	*Mystus tengera*	2.06 × 10^−04^	7.73 × 10^−05^	5.30 × 10^−03^	6.11 × 10^−05^	5.64 × 10^−03^
Silver carp	*Hypophthalmichthys molitrix*	1.69 × 10^−03^	7.42 × 10^−05^	1.87 × 10^−03^	3.97 × 10^−05^	3.67 × 10^−03^
Punti	*Punctius ticto*	1.09 × 10^−03^	2.07 × 10^−04^	7.15 × 10^−03^	1.22 × 10^−04^	8.57 × 10^−03^
Pangus	*Pangasius pangasius*	1.49 × 10^−04^	6.26 × 10^−05^	5.76 × 10^−04^	2.04 × 10^−05^	8.08 × 10^−04^
Tilapia	*Orechromis nilotichus*	3.26 × 10^−03^	6.22 × 10^−05^	1.17 × 10^−03^	1.74 × 10^−05^	4.51 × 10^−03^
Pabda	*Ompok bimaculatus*	2.09 × 10^−04^	7.01 × 10^−05^	6.96 × 10^−04^	8.33 × 10^−06^	9.83 × 10^−04^
Bele	*Glossogobius giuris*	6.37 × 10^−04^	1.46 × 10^−04^	2.43 × 10^−03^	3.63 × 10^−05^	3.25 × 10^−03^
Taki	*Channa punctatus*	3.59 × 10^−04^	6.05 × 10^−05^	1.19 × 10^−03^	8.34 × 10^−06^	1.62 × 10^−03^

#### 3.7.1 Feasible health risk and sensitivity analysis of freshwater fishes

**Fig. 5:**
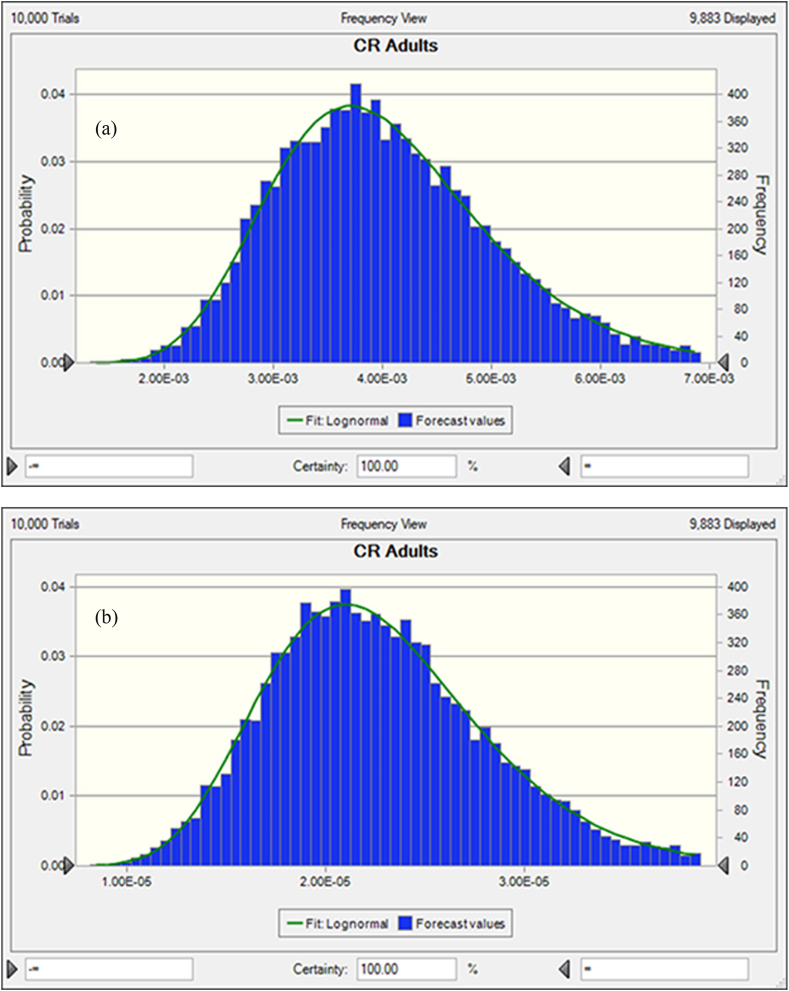

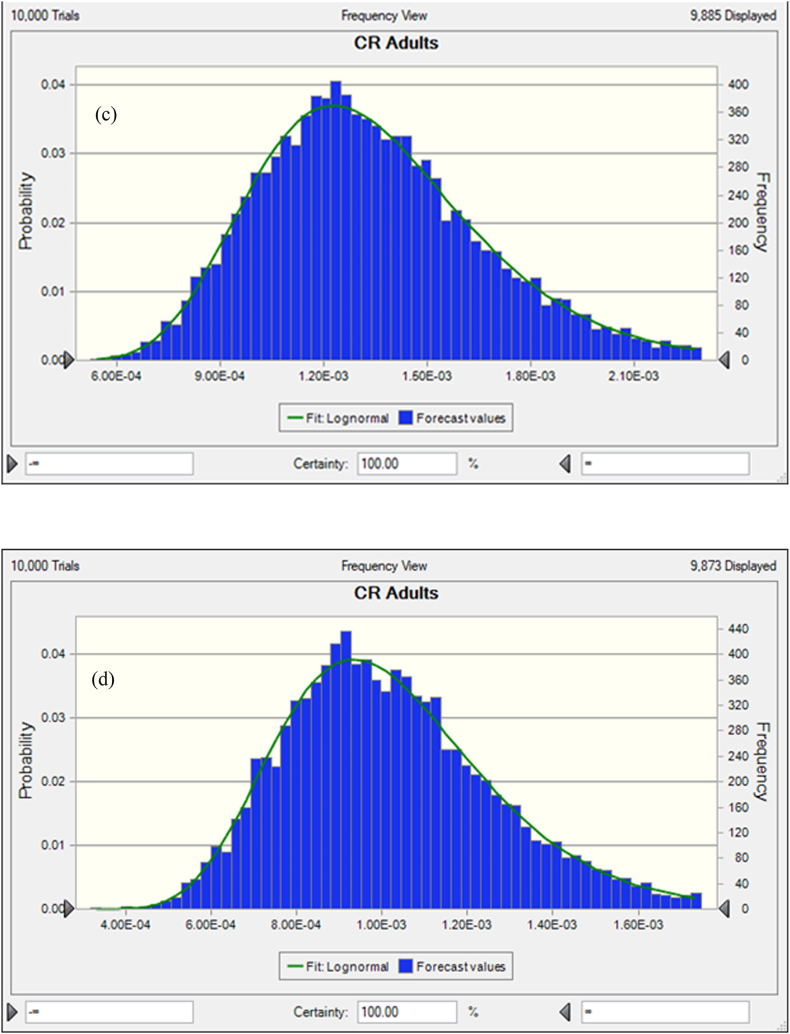
Presumed probability distribution outcomes of target cancer risk (TCR) for studied heavy metal in freshwater fishes (a) As, (b) Pb, (c) Cr and (d) Cd.

**Fig. 6:**
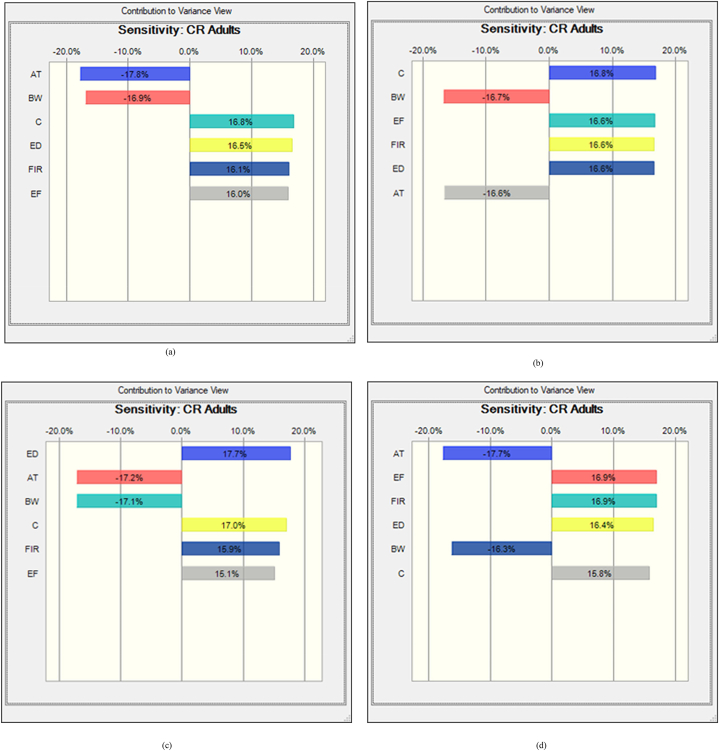
Sensitivity analysis of target cancer risk (TCR) for studied heavy metal in freshwater fishes (a) As, (b) Pb, (c) Cr and (d) Cd.

In freshwater fishes the average probability of TCR for As, Pb, Cr and Cd were 8.13E-04 ± 9.93E-04, 8.18E-05 ± 5.45E-05, 2.40E-03 ± 2.14E-03 and 3.33E-03 ± 2.41E-03, respectively (Fig. 5a, b, c, d). In this studies the percentile values were determined for freshwater fish species. The 5th and 95th percentile values of freshwater species were detected at 2.83E-03 and 7.56E-03 for As, 1.42E-05 and 5.34E-06 for Pb. On the other hand, the 5th and 95th percentile values of Cr and Cd were observed at 7.85E-04 and 3.13E-03 respectively. According to USEPA standard, the mean 5th and 95th percentile value >1E-04 is the accepted value. In our present research findings, As, Cr and Cd crossed the accepted level except the value of Pb that reported about 95% of people would be exposed to high cancer risk from the consumption of As, Cr and Cd contaminated freshwater fishes. Based on sensitivity analysis, the significance of the input variables associated to the TCR calculation was determined. For freshwater fishes, As and Pb related TCR calculation represented that C, ED, FIR and EF have positive effects with 16.8%, 16.5%, 16.1% 16.0% and 16.8%, 16.6%, 16.6%, 16,6% respectively (Fig. 6a and b). In this investigation, two variables as BW and AT have negative effects with the percentage of −16.9%, −17.8% for As and −16.7%, −16.6% for Pb were determined during TCR calculation. In the case of Chromium and Cadmium, the variables for TCR estimation C, ED, FIR and EF have positive effects with 17.0%, 17.7%, 15.1%, 15.9% and 15.8%, 16.4%, 16.9%, 16.9% respectively (Fig. 6c and d). Conversely, BW and AT have negative effects with −17.1%, −17.2% for Cr and −16.3%, −17.7% for Cd in TCR calculation. The results suggesting that metal concentrations in freshwater fishes have high risk of cancer for consumers.

### 3.8 Carcinogenic risk characterization of coastal and marine fishes

Table 8, presents the Estimated TCR for each heavy metal from the consumption of coastal and marine fishes. This study found that the TCR values for Pb and Cd were varied from 5.22 × 10^−05^ to 7.65 × 10^−05^ and 9.10 × 10^−06^ to 9.06 × 10^−05^ respectively, which indicating all the fish species are free from Pb and Cd associated cancer risk. In our present research study, 2.43 × 10^−03^ to 6.37 × 10^−03^ and 4.90 × 10^−04^ to 3.34 × 10^−03^, TCR values were documented for As and Cr respectively, that are cancerous to human health. In Sea crab, the TCR value of Cd was 5.09 × 10^−04^ [37]. In other ways, The TTCR values ranged from 3.36 × 10^−03^ to 8.40 × 10^−03^, reporting that the populations have lifetime cancer risk by the consumption of these coastal and marine fishes.

**Table 8:** Target carcinogenic risk (TCR) of metals due to consumption of coastal and marine fishes.

English name	Scientific name	TCR-As	TCR-Pb	TCR-Cr	TCR-Cd	TTCR
Parse	*Liza parsia*	6.37 × 10^−03^	5.62 × 10^−05^	1.91 × 10^−03^	4.60 × 10^−05^	8.38 × 10^−03^
Poa	*Otolithoides pama*	3.41 × 10^−03^	7.65 × 10^−05^	2.59 × 10^−03^	4.38 × 10^−05^	6.12 × 10^−03^
Marine mola	*Escualosa thoracata*	3.15 × 10^−03^	6.59 × 10^−05^	3.34 × 10^−03^	5.82 × 10^−05^	6.61 × 10^−03^
Colombo Ilish	*Hilsa ilisha*	6.07 × 10^−03^	6.59 × 10^−05^	2.17 × 10^−03^	9.06 × 10^−05^	8.40 × 10^−03^
Golden threadfin bream	*Nemipterus virgatus*	3.08 × 10^−03^	6.74 × 10^−05^	4.90 × 10^−04^	3.27 × 10^−05^	3.67 × 10^−03^
Tapshe	*Polynemus paradiseus*	5.93 × 10^−03^	6.43 × 10^−05^	6.37 × 10^−04^	2.35 × 10^−05^	6.65 × 10^−03^
Tulordandi	*Sillaginopsis panijus*	2.64 × 10^−03^	5.69 × 10^−05^	6.44 × 10^−04^	2.05 × 10^−05^	3.36 × 10^−03^
Rupchanda	*Pampus chinensis*	2.43 × 10^−03^	5.67 × 10^−05^	1.06 × 10^−03^	1.97 × 10^−05^	3.57 × 10^−03^
Mullet	*Mugil cephalus*	2.99 × 10^−03^	5.22 × 10^−05^	6.78 × 10^−04^	9.10 × 10^−06^	3.73 × 10^−03^
Tuna	*Thunnus thynnus*	3.59 × 10^−03^	5.58 × 10^−05^	8.97 × 10^−04^	3.49 × 10^−05^	4.58 × 10^−03^

#### 3.8.1 Feasible health risk and sensitivity analysis of coastal and marine fishes

**Fig. 7:**
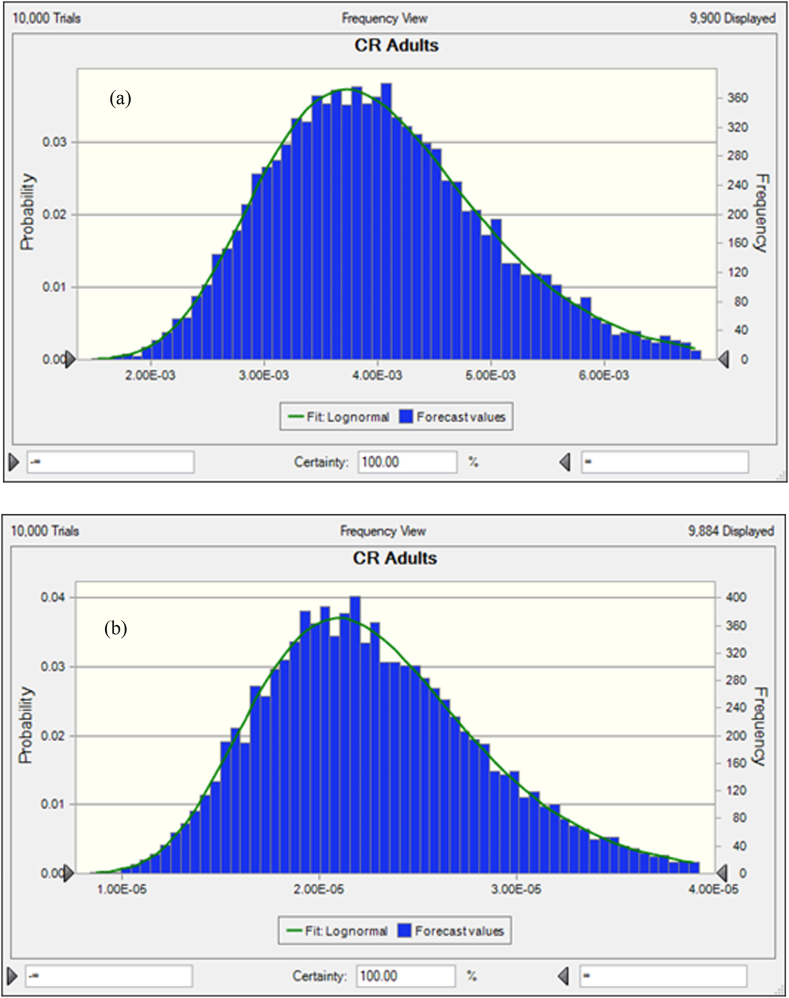

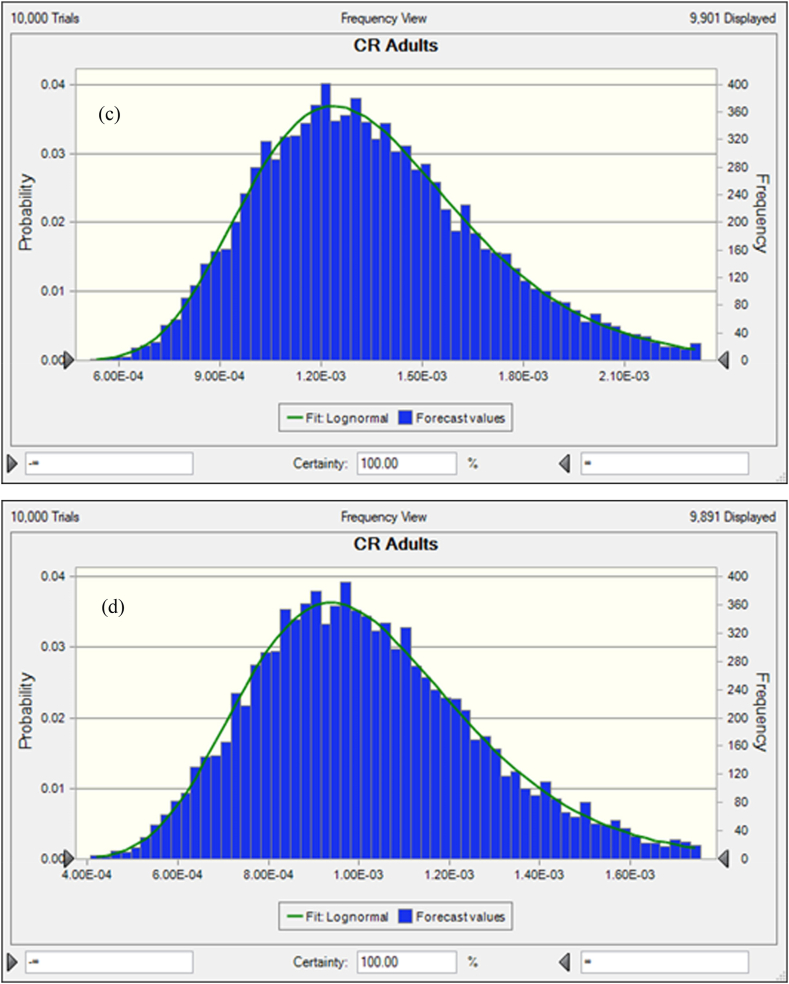
Presumed probability distribution outcomes of target cancer risk (TCR) for studied heavy metal in coastal and marine fishes (a) As, (b) Pb, (c) Cr and (d) Cd.

**Fig. 8:**
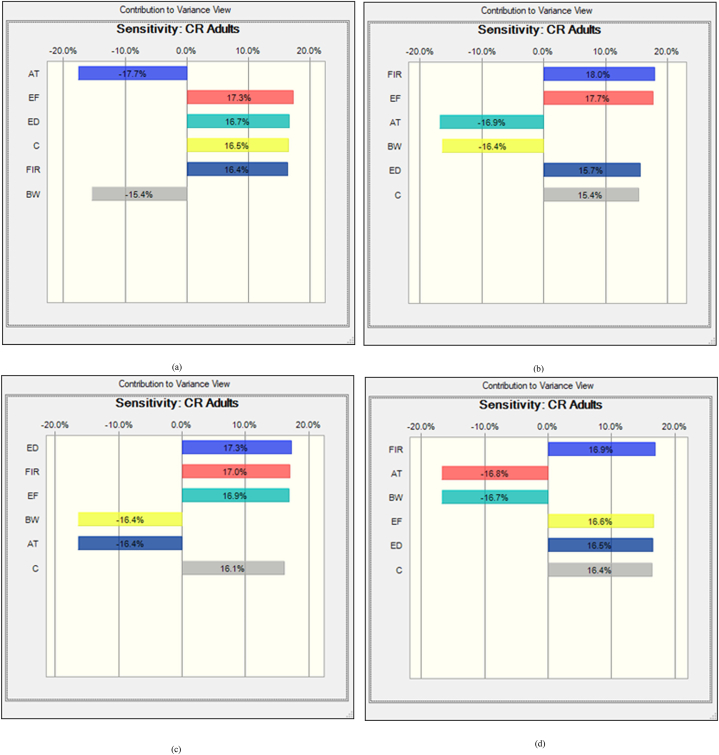
Sensitivity analysis of target cancer risk (TCR) for studied heavy metal in coastal and marine fishes (a) As, (b) Pb, (c) Cr and (d) Cd.

The average probability of TCR for coastal and marine species were shown in Fig. 7a, b, c, and d. The results represented that, and 3.97E-03 ± 1.53E-03 and 6.18E-05 ± 7.4301E-06 were the average probability of TCR for As and Pb, respectively (Fig. 7a and b). Besides, 1.44E-03 ± 9.94E-04 and 3.79E-05 ± 2.356E-05 were the average probability for Cr and Cd, respectively. The percentile values were also evaluated for coastal and marine fishes (8a, b, c and d). The 5th and 95th percentile values were recorded at 2.88E-03, 7.64E-03 for As and 1.98E-05, 3.87E-05 for Pb. However, for Cr and Cd, 5th and 95th percentile values were detected at 7.32E-04, 4.34E-03 and 5.68E-04, 3.13E-03 respectively. The percentile values for As and Cr were surpassed the recommended level (>1E-04) except the value of Pb and Cd, which denoted, 95% of coastal and marine fish consumer would be affected by As and Cr associated cancer disease. In the present research work, the significance of the input variables relevant to TCR determination were evaluated by sensitivity analysis. In the case of coastal and marine fishes, C, ED, FIR and EF have positive effects with the percentage of 16.5%, 16.7%, 16.4%, 17.3% and 15.4%, 15.7%, 18.0%, 17.7% for TCR calculation of As and Pb, respectively (Fig. 8a and b). Then again, the variables BW and AT have negative effects with −15.4%, 17.7% for As and −16.4%, −16.9% for Pb. In this study, C, ED, FIR and EF variables for TCR evaluation of Cr and Cd have positive effects with 16.1%, 17.3%, 17.0%, 16.9% and 16.4%, 16.5%, 16.9%, 16.6% respectively (Fig. 8c and d). On the contrary of the two variables as BW and AT for Cr and Cd in TCR estimation were −16.4%, −16.4% and −16.7%, −16.8% respectively. Our present findings suggested that metal concentrations of As and Cr would have cancer risk to the human being.

## 4 Conclusion

Evaluation of heavy metal concentration in fishes is necessary because fishes have good protein sources. The analysis revealed that freshwater, coastal and marine fishes in south west region were contaminated with heavy metal concentrations (Cr, Fe, Cu, As, Cd and Pb).Considering the findings from this study, the metal levels of Fe, Pb, and Cr in freshwater fishes, and As, Fe, Pb, and Cr in coastal and marine fishes, were found to surpass the maximum allowable concentration (MAC). The results of the study indicate that all fish species analyzed exhibited THQ_Fe_, THQ_Pb_, THQ_As_ and THQ_Cd_ values exceeding the safe limit (THQ>1), posing potential health risks to consumers. Additionally, the Hazard Index (HI) for both fish samples surpassed the USEPA permitted risk level (HI > 1), further highlighting the concerning health implications associated with their consumption. Moreover, the target carcinogenic risk values for As and Cr exceeded the USEPA standard limit (TCR> 1E-04), signifying that continuous consumption of these studied fishes may lead unsuitable for human consumers.

## Data availability statement

Data included in the article/supplementary material/referenced in article.

## CRediT authorship contribution statement

**Anusree Biswas:** Writing – original draft, Conceptualization. **Kaniz Fatema Kanon:** Conceptualization. **Md Anisur Rahman:** Data curation, Formal analysis. **Mohammad Shafiqul Alam:** Data curation, Formal analysis. **Sudipta Ghosh:** Methodology. **Md Almamun Farid:** Methodology.

## Declaration of competing interest

The authors declare that they have no known competing financial interests or personal relationships that could have appeared to influence the work reported in this paper.
